# Gas Exchange Disturbances Regulate Alveolar Fluid Clearance during Acute Lung Injury

**DOI:** 10.3389/fimmu.2017.00757

**Published:** 2017-07-04

**Authors:** István Vadász, Jacob I. Sznajder

**Affiliations:** ^1^Department of Internal Medicine, Justus Liebig University, Universities of Giessen and Marburg Lung Center, Giessen, Germany; ^2^Division of Pulmonary and Critical Care Medicine, Feinberg School of Medicine, Northwestern University, Chicago, IL, United States

**Keywords:** hypoxia, hypercapnia, alveolar fluid clearance, Na,K-ATPase, epithelial Na^+^ channel, acute respiratory distress syndrome, acute lung injury

## Abstract

Disruption of the alveolar–capillary barrier and accumulation of pulmonary edema, if not resolved, result in poor alveolar gas exchange leading to hypoxia and hypercapnia, which are hallmarks of acute lung injury and the acute respiratory distress syndrome (ARDS). Alveolar fluid clearance (AFC) is a major function of the alveolar epithelium and is mediated by the concerted action of apically-located Na^+^ channels [epithelial Na^+^ channel (ENaC)] and the basolateral Na,K-ATPase driving vectorial Na^+^ transport. Importantly, those patients with ARDS who cannot clear alveolar edema efficiently have worse outcomes. While hypoxia can be improved in most cases by O_2_ supplementation and mechanical ventilation, the use of lung protective ventilation settings can lead to further CO_2_ retention. Whether the increase in CO_2_ concentrations has deleterious or beneficial effects have been a topic of significant controversy. Of note, both low O_2_ and elevated CO_2_ levels are sensed by the alveolar epithelium and by distinct and specific molecular mechanisms impair the function of the Na,K-ATPase and ENaC thereby inhibiting AFC and leading to persistence of alveolar edema. This review discusses recent discoveries on the sensing and signaling events initiated by hypoxia and hypercapnia and the relevance of these results in identification of potential novel therapeutic targets in the treatment of ARDS.

A major function of the alveolar epithelium is to maintain alveolar fluid balance resulting in minimal epithelial lining fluid, thus providing optimal gas exchange (1). However, during acute lung injury (ALI) and the clinical acute respiratory distress syndrome (ARDS) the alveolar–capillary barrier fails, which leads to flooding of the alveolar space and causes a severe impairment of gas exchange (2). It is well established that clearance of alveolar edema is markedly impaired in most patients with ARDS and that this impairment is associated with worse outcomes (3). Thus, removal of the excess alveolar fluid is of significant clinical importance. The primary mechanism driving fluid reabsorption from the alveolar space is the active vectorial flux of sodium from the airspaces into the lung interstitium and the pulmonary circulation (1). Sodium, in exchange to potassium, is pumped out of the alveolar epithelial cells (AEC) basolaterally by the Na,K-ATPase, whereas Na^+^ enters the cells apically through the amiloride-sensitive and -insensitive epithelial Na^+^ channel (ENaC) (1, 4). This vectorial sodium transport process creates an osmotic gradient that drives clearance of fluid from the alveolar space (1). Failure of the alveolar–capillary barrier function leads to alveolar edema and thus to alveolar hypoventilation, resulting in hypoxemia and often elevated CO_2_ concentrations in the blood (hypercapnia) in patients with ARDS (1, 4). This is of particular importance, as several studies have shown that these conditions are not only consequences of alveolar edema but also further exacerbate alveolar fluid dysbalance by promoting formation and inhibiting reabsorption of the edema fluid (5–7). In this review, we will focus on the mechanisms by which hypoxia and hypercapnia impair alveolar fluid clearance (AFC), concentrating on the regulation of the Na,K-ATPase and ENaC in the context of ALI.

## Role of Hypoxia in Inflammation and Alveolar Fluid Balance in ALI

Adaptation to hypoxia is critically important for cellular survival as oxygen is required for ATP synthesis in the mitochondria by oxidative phosphorylation (8). During hypoxia, production of ATP is reduced by inhibition of the electron transport chain. In order to reduce energy consumption, protein translation is down-regulated and various processes with high energy demand are inhibited (8). In the context of ALI/ARDS, alveolar hypoxia and systemic hypoxemia occur as the inflamed/injured alveolar–capillary barrier fails. It is generally accepted that hypoxia is intimately coupled to inflammatory states in various organs (9). For example, inflammatory hypoxia, a manifestation of locally increased metabolism and reduced oxygen supply, may drive and further exacerbate inflammatory bowel diseases, such as ulcerative colitis or Crohn’s disease (10). While hypoxia *per se* can be an inflammatory stimulus, which up-regulates inflammatory cytokine levels, stabilization of hypoxia-inducible factor (HIF)-1α and activation of adenosine A_2A_ receptor-mediated mechanisms secondary to hypoxia may have significant anti-inflammatory effects in the lung (11, 12). Other than regulating inflammation, it is well established that hypoxia impairs alveolar fluid balance. The first preclinical studies over 15 years ago addressing the effects of hypoxia in intact rat lungs suggested that the impaired fluid balance upon exposing animals to low O_2_ levels was due to an inhibition of transepithelial sodium transport processes (5, 13). Importantly, these negative effects of hypoxia on AFC can also be observed in humans and prophylactic administration of salmeterol, a β_2_-adrenergic receptor agonist, prevents lung edema in subjects who are susceptible to high-altitude pulmonary edema, probably due to up-regulation of the Na,K-ATPase and/or ENaC (14).

## Effects of Short-Term Hypoxia on Alveolar Epithelial Na^+^ Transport

**Figure 1:**
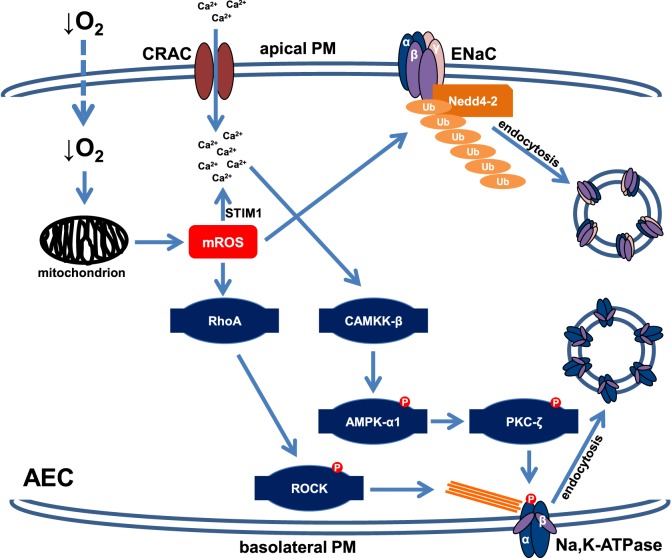
Schematic depiction of the signaling cascades impairing cell surface expression of the Na,K-ATPase and epithelial Na^+^ channel (ENaC) upon acute hypoxia. In alveolar epithelial cells (AEC), hypoxia is sensed by mitochondria, which in response release mROS. Increased mROS concentrations lead to Ca^2+^ entry through Ca^2+^ release-activated Ca^2+^ (CRAC) channels by activation of STIM1. Elevated intracellular Ca^2+^ levels result in activation of Ca^2+^/calmodulin-dependent kinase kinase (CAMKK)-β, which in turn phosphorylates and activates AMP-activated protein kinase (AMPK). Subsequently, AMPK promotes translocation of protein kinase C (PKC)-ζ to the plasma membrane (PM) where it phosphorylates the Na,K-ATPase α-subunit, thereby promoting endocytosis of the transporter. Hypoxia-induced endocytosis of the Na,K-ATPase also requires filamentous actin reorganization, which is mediated by mROS-induced activation of RhoA and ROCK. In parallel, increased mROS levels activate the E3 ubiquitin ligase Nedd4-2, which conjugates ubiquitin molecules to the ENaC β-subunit, thereby leading to endocytosis of the ENaC complex. This down-regulation of both Na,K-ATPase and ENaC cell surface expression results in impaired alveolar fluid clearance during hypoxia.

The molecular mechanisms by which hypoxia down-regulates Na^+^ transporters depend on the duration of exposure to low O_2_ levels and have been studied in various AEC lines. Severe hypoxia leads to rapid (within minutes) endocytosis of the Na,K-ATPase molecules from the plasma membrane (PM) into intracellular pools, thereby decreasing activity of the enzyme (15). It appears that in the first hour of hypoxic exposure this trafficking event is solely responsible for the hypoxia-induced impairment of Na,K-ATPase function as the total cellular abundance of the transporter remains unchanged, excluding the possibility of accelerated degradation of the transporter upon short-term hypoxia. In line with this notion, the endocytosis of the Na,K-ATPase upon hypoxia is promptly reversible upon reoxygenation (15). Furthermore, it has been reported that the effects of hypoxia on the Na,K-ATPase are mediated by mitochondrial reactive oxygen species as in ρ^0^-A549 cells, which are incapable of mitochondrial respiration, and thus unable to generate mitochondrial ROS, hypoxia does not alter the cell surface stability of the Na,K-ATPase (15, 16). Release of mitochondrial ROS upon hypoxic exposure initiates Ca^2+^ release from the endoplasmic reticulum (ER) and redistribution of the calcium sensor STIM1 to the ER PM junctions, thereby resulting in calcium entry through Ca^2+^ release-activated Ca^2+^ channels, which in turn activates Ca^2+^/calmodulin-dependent kinase kinase (CAMKK)-β, a well-known inducer of the metabolic sensor AMP-activated protein kinase (AMPK) (17). Of note, AMPK is a major regulator of cellular energy balance and activation of the kinase leads to inhibition of processes that require high energy (18); thus, playing a central role in the adaptation to hypoxia. As the Na,K-ATPase accounts for ~30–80% of the energy expenditure of cells (8), rapid down-regulation of the transporter driven by AMPK appears to be key in this adaptation process. Once activated, AMPK-α1 directly phosphorylates protein kinase C (PKC)-ζ at the Thr410 residue (19). This is of relevance as phosphorylation of PKC-ζ at Thr410 drives translocation of the protein kinase to the PM where it phosphorylates the Na,K-ATPase at Ser18. It is well documented that phosphorylation of this serine residue promotes endocytosis of the Na^+^ pump from the PM (15). In parallel, upon hypoxic exposure mitochondrial ROS activate RhoA, a member of the Rho GTPase family and its downstream effector, the Rho-associated serine/threonine kinase (ROCK), a central regulator of filamentous actin reorganization, which has been implicated in the control of endocytosis (20, 21). Thus, in the alveolar epithelium the mitochondria serve as hypoxia sensors and release of mitochondrial ROS initiates a rapid and highly specific signaling cascade that leads to endocytosis of the Na,K-ATPase from the PM and thereby alveolar epithelial dysfunction (Figure 1).

Moreover, reactive oxygen and nitrogen species (RONS) have also been implicated in the down-regulation of ENaC (22). Recently, two Tyr residues located in the extracellular loop of the α-subunit of ENaC, Tyr279 and 283, have been identified as potential targets of oxidation by RONS (23). Various additional ion channels have been shown to be modulated (mainly down-regulated) by ROS and RONS by regulation of channel transcription, direct oxidation, nitration or nitrosylation of channels, and via interference with signaling patterns regulating activity, trafficking or expression of channels (24).

## Effects of Sustained Hypoxia on the Na,K-ATPase and ENaC

Sustained hypoxia down-regulates sodium transporter function in the alveolar epithelium by at least two independent mechanisms. As discussed above, during cellular adaptation to hypoxia protein translation is down-regulated to reduce energy consumption (8). Indeed, several reports documented that upon long-term hypoxia both mRNA and total protein levels of the Na,K-ATPase and ENaC are decreased (25). A second, and more specific mechanism is the ubiquitination and directed degradation of the transporters. Ubiquitination is a post-translational modification during which ubiquitin molecules are conjugated (mostly but not exclusively) to specific lysine residues of target proteins, thereby controlling stability, function, and localization of the target (26, 27). Regarding the regulation of Na,K-ATPase upon prolonged hypoxic exposure, it has been documented that degradation of the enzyme occurs first (after approximately 2 h) in the PM, whereas exposing AEC to severe hypoxia for up to 24 h results in degradation of the Na,K-ATPase in intracellular pools (16). Considering that the Na,K-ATPase accounts for a significant proportion of the energy expenditure of cells, as mentioned above, it appears logical that as an adaptive mechanism to hypoxia the active Na,K-ATPase molecules (located at the PM) will be removed from the surface and degraded more rapidly than degradation of the inactive molecules (located in the intracellular pools) occurs to reduce cellular energy consumption and thus promote survival (8). A subsequent study established that four Lys residues (Lys16, 17, 19, and 20) surrounding the PKC-ζ phosphorylation site (Ser18) at the N-terminus of the Na,K-ATPase α-subunit are required for ubiquitin conjugation (28). Of note, phosphorylation of the Na^+^ pump by PKC-ζ at Ser18 is necessary for ubiquitination, perhaps by increasing affinity of ubiquitin to the phosphorylated target, highlighting the possibility of cross-talk between phosphorylation and ubiquitination (28). The E3 ubiquitin ligase of the Na,K-ATPase remains to be identified. Although the E3 ubiquitin ligase, von Hippel Lindau protein has been implicated in the degradation of the Na,K-ATPase upon hypoxia, it has also been shown that this E3 ligase does not directly target the Na^+^ pump (29). Further research on the E3 ligase targeting the Na,K-ATPase will be of particular importance, as that molecule may represent a highly specific druggable target of impaired AFC upon gas exchange disturbances.

Ubiquitination also plays a central role in the down-regulation of ENaC upon sustained hypoxia. It is well established that the E3 ubiquitin ligase, Nedd4-2 plays a pivotal role in the regulation of ENaC cell surface stability by directly targeting α-, β-, or γ-ENaC depending on the stimulus leading to endocytosis and/or degradation of the channel (30). It has been reported that upon hypoxic exposure for 24 h in mice carrying a truncation of the C-terminus of β-ENaC (homozygous β-Liddle mouse strain), thus preventing interactions with Nedd4-2, amiloride-sensitive AFC remains normal, whereas in wild-type mice AFC is decreased by approximately 70%. Furthermore, a marked reduction in the amiloride-sensitive apical Na^+^ current upon hypoxia can be fully prevented by inhibition of the proteasome and by the ROS scavenger *N*-acetyl-cysteine (31), suggesting that the ubiquitin–proteasome system is critically required for the hypoxia-driven down-regulation of ENaC and further confirming the central role of ROS in the hypoxic impairment of alveolar epithelial Na^+^ transport processes.

## Role of Hypercapnia in Inflammation and Alveolar Fluid Balance in ALI

While in most patients with ARDS hypoxia can be corrected by the use of mechanical ventilation with elevated inspired fractions of oxygen, hypercapnia often persists in part due to the low tidal volume ventilation strategy, which is required to minimize further ventilator-induced lung injury (32). While “protective” mechanical ventilation with low tidal volumes is clearly beneficial (33), the effects of hypercapnia in the context of lung injury remain a topic of intense debate. Several studies suggested that hypercapnia is tolerable or even beneficial whereas others documented that various aspects of the hypercapnic effects on alveolar epithelial function are deleterious, leading to the terms of permissive, therapeutic, and non-permissive hypercapnia, respectively (6, 34). It is very well established that excessive inflammation plays a central role in the pathogenesis ARDS (35). Moreover, respiratory acidosis (a decrease in the pH of the blood secondary to hypercapnia) has several anti-inflammatory properties, such as reduction of pro-inflammatory cytokines, impairment of neutrophil function, and inhibition of generation of free radicals (36). Thus, it appears logical that hypercapnia (or the associated acidosis) may be beneficial in the context of ARDS. In contrast, a recent secondary analysis of three large prospective non-interventional clinical studies recruiting mechanically ventilated patients with moderate and severe ARDS in over 900 ICUs from 40 countries documented that hypercapnia is independently associated with a markedly higher ICU mortality (37), which is further supported by another study in which hypercapnic acidosis in the first 24 h after ICU admission was associated with higher hospital mortality (38). There are several factors that may lead to worse outcomes of hypercapnic patients with ARDS. Although, and as discussed above, the hypercapnia-associated acidosis may exhibit early anti-inflammatory effects; recently, it has become increasingly evident that hypercapnia impairs innate immunity, thereby potentially increasing susceptibility of patients with ARDS to bacterial infections (39, 40). Furthermore, recent studies established that hypercapnia, independently of changes in pH, impairs alveolar epithelial fluid balance by inhibiting AFC, and thus resolution of pulmonary edema (41–43). As it is well documented that clearance of the excess, protein-rich alveolar edema in patients with ARDS is critical for survival, this aspect is of clinical relevance.

## Effects of Acute Hypercapnia on the Na,K-ATPase and ENaC

**Figure 2:**
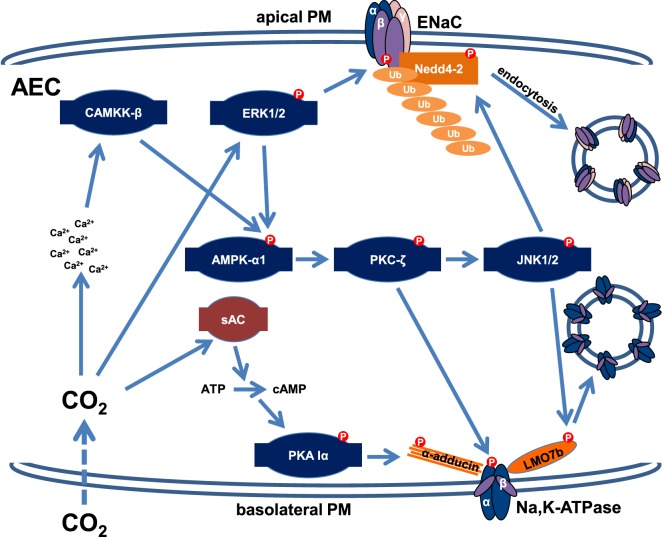
Schematic representation of the signaling signatures down-regulating the Na,K-ATPase and epithelial Na^+^ channel (ENaC) upon acute hypercapnia. Hypercapnia leads to phosphorylation and subsequent endocytosis of Na,K-ATPase by an AMP-activated protein kinase (AMPK)-dependent and an AMPK-independent mechanism. An acute elevation in CO_2_ levels in alveolar epithelial cells (AEC) leads to an increased intracellular Ca^2+^ concentration by a yet unidentified mechanism. A subsequent activation of the Ca^2+^/calmodulin-dependent kinase kinase (CAMKK)-β/AMPK-α/protein kinase C (PKC)-ζ signaling cascade results in phosphorylation of the Na,K-ATPase α-subunit. PKC-ζ also activates c-Jun N-terminal kinase (JNK), which phosphorylates the scaffolding protein LMO7b, thereby promoting endocytosis of the Na,K-ATPase. Furthermore, elevated CO_2_ is sensed by the sAC, which in turn activates protein kinase A (PKA) type Iα by cAMP in microdomains at close proximity of the basolateral membrane, resulting in phosphorylation of α-adducin, which is required for the rearrangement of the actin cytoskeleton necessary for endocytosis. Moreover, CO_2_ activates extracellular signal-regulated kinase (ERK), which is also required for AMPK stimulation. ERK phosphorylates the ENaC β-subunit, thereby attracting the E3 ubiquitin ligase Nedd4-2, which is phosphorylated and activated by JNK upon hypercapnic exposure, leading to polyubiquitination of β-ENaC, and a reduction of ENaC abundance at the apical PM. Collectively, these mechanisms impair the function of both the Na,K-ATPase and ENaC and are responsible for the hypercapnia-induced inhibition of alveolar edema clearance.

Because elevated CO_2_ levels impair AFC within minutes, it has been hypothesized that much like variations in oxygen concentration, levels of CO_2_ may be sensed by the alveolar epithelium (41, 43–45). It was described several decades ago that excitable cells, such as specialized brainstem neurons or the glomus cells of the carotid body serve as central and peripheral chemoreceptors of CO_2_ and depolarize upon hypercapnia (46). In contrast, only recently it became evident that elevated CO_2_ levels also initiate specific signaling patterns in non-excitable cell types, such as the alveolar epithelium, independently of intra- or extracellular pH, carbonic anhydrases, or ROS (41, 42). Most recently, the hemichannel connexin 26 has been implicated in CO_2_ sensing (47). Interestingly, the high CO_2_-induced signaling leads to a rapid down-regulation of the Na,K-ATPase activity, thereby inhibiting AFC, one of the major functions of the alveolar epithelium (41, 42). This hypercapnia-induced signaling pattern has been dissected in the past years and we now know that elevated CO_2_ levels increase intracellular Ca^2+^ concentrations within seconds leading to activation of CAMKK-β, which stimulates the metabolic sensor AMPK. Similarly to the effects of hypoxic exposure, the hypercapnia-induced activation of AMPK leads to translocation of PKC-ζ to the PM, where the kinase phosphorylates the Na,K-ATPase α-subunit, thereby promoting endocytosis of the transporter from the PM (41, 43). The endocytosis of the Na,K-ATPase also requires activation of the c-Jun N-terminal kinase (JNK), which is similarly to PKC-ζ also downstream of AMPK in the CO_2_-induced signaling cascade (48). Upon hypercapnia, activated JNK phosphorylates the scaffolding protein LMO7b at the Ser1295 residue, which enables interaction of the scaffolding protein with the Na,K-ATPase at the PM of AEC, thereby promoting endocytosis and thus inhibition of the transporter (49). Of note, the requirement of JNK in the hypercapnia-induced inhibition of the Na,K-ATPase was not only shown in mice, rats, and human cells but also in *Drosophila melanogaster*, suggesting that at least some elements of the CO_2_-induced signaling pattern are evolutionarily conserved (48). Interestingly, this pathway overlaps with that of initiated by acute hypoxia; however, the effects of hypercapnia are independent of mitochondrial ROS. Furthermore, unlike in hypoxia the source of Ca^2+^ upon hypercapnic exposure of the alveolar epithelium remains unknown and the regulation of the endocytic machinery appears to be different in hypoxia and hypercapnia, where activation of RhoA and ROCK as opposed to JNK and LMO7b are required, respectively (Figure 2). Moreover, some effects of hypercapnia and hypoxia are opposing, as elevated CO_2_ levels inhibit the HIF-driven adaptation mechanisms to hypoxia (50). Recently, an alternative and AMPK-independent pathway has also been identified in the elevated CO_2_-induced down-regulation of the Na,K-ATPase. It has been reported that hypercapnia also activates the recently identified metabolic sensor CO_2_/HCO_3_^−^ responsive soluble adenylyl cyclase (CO_2_/HCO_3_-sAC), which by producing cAMP in specific microdomains in the proximity of the PM led to activation of protein kinase A (PKA) type Iα that phosphorylated the actin cytoskeleton component α-adducin at Ser726, thereby promoting endocytosis of the Na,K-ATPase (51). This novel pathway is AMPK-independent as AMPK phosphorylation upon hypercapnia also occurs in the presence of an siRNA against CO_2_/HCO_3_-sAC, similarly to PKA activation in AEC after AMPK silencing, suggesting that both pathways are required for the hypercapnia-induced down-regulation of Na,K-ATPase cell surface stability.

Most recently, the molecular mechanism impairing ENaC cell surface stability in AEC upon acute hypercapnic exposure has been described (52). Upon hypercapnia, extracellular signal-regulated kinase (ERK), which has been previously identified in the CO_2_-induced signaling pattern as an activator of AMPK (53), directly phosphorylates the ENaC β-subunit at Thr615. Moreover, JNK, which is activated by AMPK upon activation of the latter kinase by ERK, phosphorylates Nedd4-2 at Thr899, thereby increasing the activity of the E3 ubiquitin ligase (52). These phosphorylation events promote the interaction of β-ENaC and Nedd4-2 and lead to polyubiquitination of β-ENaC and subsequent endocytosis of the ENaC complex, thereby reducing cell surface stability of the channel.

## Effects of Sustained Hypercapnia on Alveolar Epithelial Na^+^ Transport and Repair

Interestingly, the effects of long-term elevated CO_2_ levels on the Na,K-ATPase are reversible. Exposing rats to elevated CO_2_ concentrations for up to 1 week leads to a sustained and marked decrease in AFC (43). Similarly, exposure of AEC to elevated CO_2_ levels for up to 24 h causes a sustained reduction of Na,K-ATPase abundance at the PM (43). However, when exposing rat lungs to normocapnia after a hypercapnic treatment for 1 h, levels of AFC rapidly return to normal (41). Furthermore, treatment of rat lungs with the β-adrenergic receptor agonist, isoproterenol not only prevents but also reverses the hypercapnia-induced decrease in AFC, confirming that the high CO_2_-induced AFC impairment is reversible at least in the first hour of hypercapnia (43). Moreover, sustained hypercapnia induces the microRNA, miR-183, which down-regulates isocitrate dehydrogenase 2, an enzyme that catalyzes the conversion of isocitrate to α-ketoglutarate during the tricarboxylic acid cycle (54). This effect leads to mitochondrial dysfunction thus, inhibiting proliferation of AEC, which may impair repair mechanisms and resolution of lung injury.

## Conclusion

Gas exchange disturbances are hallmarks of ALI and ARDS. Both low O_2_ and elevated CO_2_ levels are rapidly sensed by the alveolar epithelium, the site of oxygen uptake and CO_2_ elimination, leading to adaptation but also deleterious effects on cellular function. Both hypoxia and hypercapnia are intimately coupled to inflammation and by highly specific and partially described signaling pathways, which inhibit epithelial sodium transport processes impair AFC. As alveolar hypoxia and hypercapnia cannot always be corrected at the areas of severe injury, interfering with these deleterious signals may lead to novel therapies against ARDS.

## Author Contributions

IV: drafting the work and preparing figures. JS and IV: revising it critically for important intellectual content. Both authors approved the final version of the manuscript.

## Conflict of Interest Statement

The authors declare that the research was conducted in the absence of any commercial or financial relationships that could be construed as a potential conflict of interest.
